# Facial expression recognition (FER) survey: a vision, architectural elements, and future directions

**DOI:** 10.7717/peerj-cs.2024

**Published:** 2024-06-03

**Authors:** Sana Ullah, Jie Ou, Yuanlun Xie, Wenhong Tian

**Affiliations:** School of Information and Software Engineering, University of Electronic Science and Technology of China, Chengdu, Sichuan, China

**Keywords:** Computer vision, Emotion recognition technology, Basic & compound emotions, Cloud computing, Internet of Things (IoT)

## Abstract

With the cutting-edge advancements in computer vision, facial expression recognition (FER) is an active research area due to its broad practical applications. It has been utilized in various fields, including education, advertising and marketing, entertainment and gaming, health, and transportation. The facial expression recognition-based systems are rapidly evolving due to new challenges, and significant research studies have been conducted on both basic and compound facial expressions of emotions; however, measuring emotions is challenging. Fueled by the recent advancements and challenges to the FER systems, in this article, we have discussed the basics of FER and architectural elements, FER applications and use-cases, FER-based global leading companies, interconnection between FER, Internet of Things (IoT) and Cloud computing, summarize open challenges in-depth to FER technologies, and future directions through utilizing Preferred Reporting Items for Systematic reviews and Meta Analyses Method (PRISMA). In the end, the conclusion and future thoughts are discussed. By overcoming the identified challenges and future directions in this research study, researchers will revolutionize the discipline of facial expression recognition in the future.

## Introduction

Emotions are important components of our lives, based on which we make numerous life decisions. Facial expression recognition (FER) is critical for computer games, recommendation systems, human–computer interaction, stress control, mental health monitoring, autism monitoring, and many more. Facial expression recognition is crucial for understanding human intentions and emotions, facilitating effective human-machine interaction and communication. Communication is a critical tool in promoting societies to permit humans to adapt, progress, and survive. It is the most powerful signal through which humans exchange intentions and emotional states, and 55% of the communication is performed through facial expressions (Saganowski, 2022; Haryanto & Agastya, 2022). Commonly, emotions and intentions are conveyed by humans either through involuntary language, facial expression, and gestures. Based on this, various FER systems have been designed to address real-world challenges such as driver safety, human-machine interaction, medical assessment, and lie detection (Appasaheb Borgalli & Surve, 2023). Facial expression recognition technology has applications in different fields, such as access control, law enforcement, entertainment & gaming, transportation, security & surveillance, advertisement & marketing, health & safety, education, finance & banking, and many more. Emotions are divided into two categories, namely basic and compound emotions. The seven basic emotions include disgust, anger, neutrality, sadness, fear, surprise, and happiness. Compound emotions, such as ‘happily surprised,’ are formed by combining basic emotions, representing the simultaneous appearance of two emotions on a human face. Researchers have worked on basic emotions, but recognizing compound emotions is challenging due to their complex features. Image recognition technology utilizes computers to understand, analyze, and process diverse patterns of objects and targets (Huo, Yu & Liu, 2023). The evolution of convolutional neural networks (CNNs) revolutionized computer vision. Diverse CNN models have been applied to computer vision tasks like segmentation, object detection, and image recognition. CNNs are popular because they can automatically learn features from an image. Different filters are used in the convolutional layers, and techniques such as pooling are employed to reduce dimensions and increase robustness against variations. With the rapid evolution of technology, different methods have been used at different times to recognize emotions. Traditional feature extraction-based methods, including LBP, HOG, NMF, K-NN, Random Forest, and SVM, were utilized in the early stages. These methods were effective for selected tasks, and manual feature engineering was time-consuming and inefficient for complex image classification (Mohammadi & Kalhor, 2021; Dargan et al., 2020; Jiao & Zhao, 2019; Mahesh, 2020).

The integration of deep learning methods like CNNs, recurrent neural networks (RNNs), transfer learning, Siamese networks, and generative adversarial networks (GANs) addressed several limitations of traditional machine learning methods. Furthermore, different attention mechanisms were introduced further to improve the performance of deep learning methods, and deep learning methods outperformed the traditional machine learning methods in image recognition and classification. Despite their benefits, attention mechanisms integrated into deep learning methods for improving performance across diverse tasks still struggle with capturing contextual information in data and managing long-range dependencies (Chen et al., 2021; Mathew, Amudha & Sivakumari, 2021; Kunapuli, 2023; Diniz et al., 2021).

A new type of neural network, the transformer, was introduced, which utilized a self-attention mechanism to extract intrinsic features and showed potential performance in many applications of AI (Mazzia et al., 2022; Han et al., 2022). When applied for the first time, the transformer achieved significant performance in many tasks related to language processing (NLP). With the strong representation capabilities and achieving significant breakthroughs by the transformer-based models without any fine-tuning in the NLP, the researchers are now inspired and applied transformer-based models to computer vision tasks. CNNs are counted as a fundamental component, but currently, transformers are utilized as the best hybrid component with CNNs for better performance. Digital images are complex and contain high-level information like patterns, scenes, and objects. The information in digital images can be extracted and analyzed through vision-based algorithms and models; the important insights about the image include extracting features, tracking movements, and recognizing objects. Computer vision is a critical area of research, as its applications extend across nearly all fields. However, extracting high-level meaningful information from an image of compound facial expressions of emotion is challenging for researchers due to background clutter, pose, and brightness variations (Xie, Tian & Ma, 2020; Tunstall, Werra & Wolf, 2022; Chitty-Venkata et al., 2022).

This article presents the current trends in facial expressions of emotion technologies research, propelled by applications and the need for convergence in several interdisciplinary technologies. The main contributions and novelties of this article are as follows:

(1) We provide a comprehensive overview of the future vision of facial expression recognition technology and discuss the cutting-edge technologies that will enable its realization. This forward-looking perspective sets the stage for the advancements presented in the article (section ‘Research methodology’).

(2) We offer clear and concise definitions of related terminologies, elements, and trends in face recognition technologies. Furthermore, we have discussed various models and algorithms utilized in FER. By clarifying these key concepts, we establish a solid foundation for understanding the state-of-the-art in this field (section ‘Facial expression recognition technology in the next decade’).

(3) We present a wide range of applications for facial expression recognition technologies and highlight the top companies around the globe that are leading the way in this domain. This industry-focused analysis demonstrates the practical relevance and potential impact of our research (section ‘Facial expression recognition models/frameworks’).

(4) We delve into the interconnection among cloud computing technology, Internet of Things (IoT), and facial expression recognition technologies, providing a detailed discussion on how these technologies converge to enable powerful new applications. This interdisciplinary perspective is a unique contribution of our article (section ‘Facial expression recognition, Internet of Things (IoT), and cloud computing’).

(5) We identify and analyze the open challenges faced by facial expression recognition technologies and propose future research directions to address these challenges. By highlighting these research gaps and opportunities, we set the agenda for advancing the field (section ‘Research methodologies issues’).

(6) We conclude the article with a summary of our findings and offer insightful thoughts on the future of facial expression recognition technologies. This synthesis and forward-looking perspective distinguish our work from existing literature in the field (section ‘Conclusions’).

By presenting these unique contributions and novelties, our article stands out in the crowded field of facial expression recognition research. The comprehensive overview, interdisciplinary analysis, and future-oriented perspective make this work a valuable resource for researchers and practitioners alike.

### Research methodology

**Figure 1:**
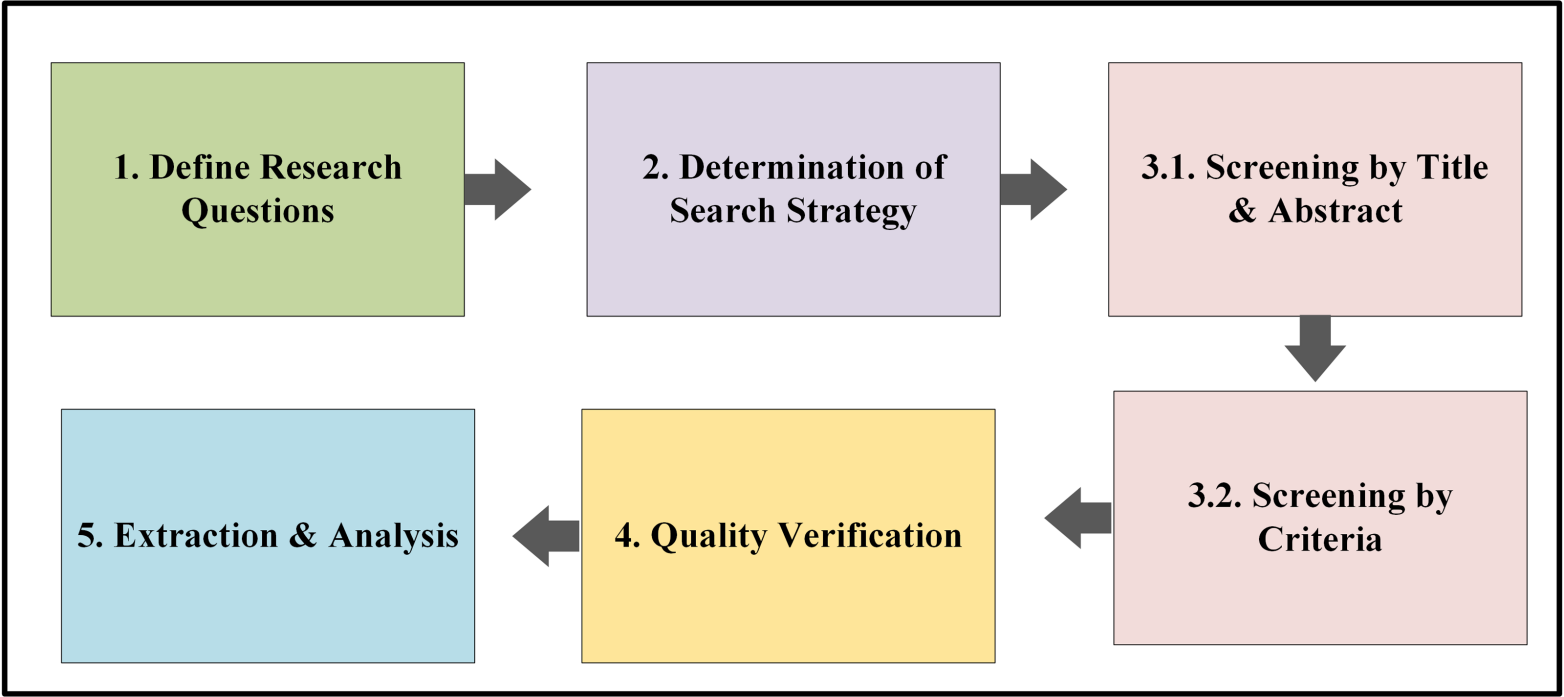
PRISMA review protocol.

We have utilized the Preferred Reporting Items for Systematic reviews and Meta-Analyses Method (PRISMA); review protocols are developed based on PRISMA guidelines, shown in Fig. 1. Based on PRISMA guidelines, five steps have been followed: (1) defining research questions (RQ), (2) conducting a comprehensive literature search using predefined keywords and databases, (3) screening and selecting articles based on predefined inclusion and exclusion criteria, (4) assessing the quality of selected articles, and (5) extracting and analyzing data from the included studies. This systematic approach ensures the transparency, reproducibility, and credibility of the review process (Page et al., 2021; Lu et al., 2024).

### Research questions

Considering the research objectives, the following key research questions are formulated:

RQ 1: What will facial expression recognition technology be like in the next decade, and what about the recent trends in face recognition technology?

RQ 2: What are the standard architectural elements of the facial expression recognition system?

RQ 3: What are the different models/algorithms/architectures used in facial expression recognition?

RQ 4: What are the broad applications of face recognition technology in various fields, and what about the world’s top FER technologies companies?

RQ 5: What is the interconnection between FER, the IoT, and cloud computing?

RQ 6: What are the open challenges to FER technology in integrating into various real-world fields?

RQ 7: What are the limitations and challenges concerning FER technology and the future direction for new research in the field of computer vision?

Table 1 shows the search strings with mapping of PICO elements for each request question.

**Table 1:** Search strings with mapping of PICO elements.

**Research question**	**Population**	**Interest**	**Comparison**	**Outcome**	**Search string**
What will facial expression recognition (FER) technology be like in the next decade, and what about the recent trends in face recognition technology?	Humans or machines	FER in future, trends of FER	No specific comparison group required	Future of FER, recent trends of FER	(“facial expression recognition” OR “emotion recognition from faces”) AND (“FER trends”)
What are the standard architectural elements of the facial expression recognition system?	*	FER architectural elements	*	FER architectural elements	(“Standard FER architecture” OR “FER architecture elements”)
What are the different models/algorithms/architectures used in facial expression recognition?	*	FER models and architectures	Comparison among FER architectures/Models	FER Models/architectures	(“FER architecture” OR “FER models” “FER algorithm”)
What are the broad applications of FER technology in various fields, and what about the world’s top FER technologies companies?	Humans or machines or company	FER applications, FER tech companies	No specific comparison group required	FER applications, FER tech companies	(“FER application” OR “FER technology companies”)
What is the interconnection between FER, the internet of things (IoT), and cloud computing (CC)?	Humans or machines	FER, IoT, CC	*	Interconnection among FER, IoT & CC	(“FER” AND “IoT” AND “CC”)
What are the open challenges to FER technology in integrating into various real-world fields?	*	FER challenges in diverse domains	*	Extract FER challenges in diverse domains	(“FER challenges OR “FER limitations”)
What are the limitations and challenges concerning FER technology and the future direction for new research in the field of computer vision?	*	FER technology challenges, FER future directions	*	Extract FER challenges & future directions	(“FER challenges OR ”FER future directions”)

### Strategy for searching articles

Searching for relevant research articles from online libraries is an important task. We designed search strings according to SLR guidelines (Kitchenham et al., 2009) and selected six libraries for article searching: IEEE Xplore, ACM Digital Library, Scopus, Web of Sciences, ScienceDirect, Springer, and Google Scholar. The keywords and their synonyms were derived from our research questions (Section ‘Strategy for searching articles’) and included terms such as “facial expression recognition”, “emotion recognition”, “deep learning”, “computer vision”, and “IoT”. We conducted both forward and backward snowballing to identify additional relevant articles.

We screened around 3,245 articles from the target databases according to the guidelines of PRISMA. The details of the number of articles screened from different libraries through the listed keywords used for searching in each library are shown in Table 2.

**Table 2:** Summary of search strategy and results.

Database	Keywords	Number of papers
ACM Digital Library	Facial expression recognition, emotion recognition, deep learning, computer vision, IoT, compound emotions,	708
IEEE Xplore	*	230
ScienceDirect,	*	821
Springer	*	146
Google Scholar	*	720
Scopus	*	357
Web of Science	*	263

### Criteria for article selection

In the article selection criteria, we followed two phases: primary selection and secondary selection. The primary selection was based on title, keywords, and abstract screening. In the secondary selection phase, we applied the following inclusion and exclusion criteria:

Inclusion criteria:

1. Peer-reviewed conference papers and journal articles

2. English-language articles

3. Studies focusing on facial expression recognition, emotion recognition, deep learning, and computer vision

Exclusion criteria:

1. Non-peer-reviewed articles, such as preprints, theses, and dissertations

2. Articles written in languages other than English

3. Studies focusing on synthetic image generation techniques and other unrelated topics

4. Duplicate articles.

### Quality assessments of articles

Apart from the previously followed inclusion and exclusion criteria, we have analyzed each article based on its quality, content, and publication venue.

### Data extraction and synthesis

We extracted different information from the selected articles based on our research questions. By following the the guidelines of PRISMA for article selection criteria and articles quality assessment, we have extracted results based on 141 articles.

### Facial Expression Recognition Technology in the Next Decade

Facial expression recognition technology has a rich history, which evolved over decades. From the 1960s till the present, it has significantly evolved. In the early 1960s, the researchers were more focused on developing algorithms for basic features like mouth, nose, and eye for identification and analysis from photographs. Andrejevic & Selwyn (2022) developed a system for extracting certain photo features.

In the 1980s and 1990s, the Eigenfaces techniques were developed to recognize facial expressions of emotion. It was working based on the principal component analysis (PCA) approach. Other methods, such as sparse representation, manifold learning, and linear subspace, were also developed for facial recognition. In this period, most methods were features-based, like the Elastic Bunch Graph Matching (EBGM) algorithm and the Active Appearance Model (AAM) (Turk & Pentland, 1991a; Turk & Pentland, 1991b).

In the years following 2,000, local feature-based methods like local binary patterns (LBP) and Gabor features started gaining popularity in computer vision for face recognition. In this era, machine learning and statistical methods such as hidden Markov models (HMM) and support vector machines (SVM) were introduced for robust and accurate identification. In the 2010s and onwards, the deep learning-based models era started. The convolutional neural networks (CNNs) models were introduced, and later, in the 2012s, the deep learning-based models gained more popularity due to the success of AlexNet in the ImageNet competition (Xue-fang & Tao, 2013; Krizhevsky, Sutskever & Hinton, 2012; Taskiran, Kahraman & Erdem, 2020).

There is more research on basic emotions but less on compound emotion recognition, and researchers have been working more recently on compound emotion recognition. Most of the recently introduced compound emotions combine two basic emotions. However, compound emotion can be the combination of more than two basic emotions, so future research on compound emotion will be considered and observed. The compound emotions introduced a few, and new compound emotions will be introduced in future research. The recognition is based on visual modality, and it will be better to introduce more modalities of detailed and complementary feature extraction (Li & Li, 2020).

The perception and production of compound emotion categories open a new level of complexity in computer vision for human–computer interfaces, designing computer vision, social communication, and human cognition for researchers. The important areas of researchers’ interest in compound emotion recognition are cognitive impairments and psychiatric disorders. It is also the point of investigation for researchers in future research on which the researchers have focused on whether the cognitive processes and cognitive representation are the same for compound emotions and basic emotions or these are different for basic and compound emotions (Du, Tao & Martinez, 2014; Martinez & Du, 2012).

The latest research in computer vision shows that emotional features and emotions change from culture to culture. Computer scientists have focused on developing deep learning models to overcome cultural issues in emotional features due to culture. The cultural evolutionary research on emotions is in the early stage, and researchers are developing strategies and methods for basic and compound emotion recognition through applying evolutionary hypotheses in emotions study (Jack et al., 2012; Lindquist et al., 2022; Brooks et al., 2023).

Novel deep learning models will be developed while considering inta-class variations, including noise, age variations, illumination variations, pose, and occlusion. The deep learning models will be evaluated on the datasets with diverse images. New deep-learning approaches will be developed for real-time emotion recognition, which will detect emotions for untrained classes in the future (Kuruvayil & Palaniswamy, 2022).

Numerous research works have been performed on facial expression recognition and body pose, and some methods have been performed efficiently in specific settings. However, their performance could be better in unconstrained and natural real-life environments. In the future, the researchers will be focusing on developing new models while considering the combined information of the person (body bounding box) and scene context information (whole image) for better performance in natural and unconstrained environments (Kosti et al., 2019).

The facial expressions of emotion changed with changes in gender, culture, and age. An ideal perfect dataset contains images to facilitate cross-cultural, cross-gender, and cross-age research through deep learning methods. The research on pose variant issues and occlusion in emotion recognition received less attention from researchers due to the unavailability of a large dataset having head-pose and occlusion annotations. The researchers are trying to create a dataset that fulfills the above mentioned issues with images of complex scenarios and natural environments instead of controlled ones. Some emotions are created through the combination of basic emotions with complex features. To properly recognize compound emotions, there is a need for a novel deep learning model to extract compound emotion features appropriately (Benitez-Quiroz, Srinivasan & Martinez, 2016; Barsoum et al., 2016; Li & Deng, 2020).

Diverse facial expression recognition algorithms have been developed for practical and theoretical applications. Recently, the researchers’ focus shifted from a controlled environment to challenging wild environmental conditions. There are two main challenges in the wild environmental conditions, including pose variation and occlusion. Some of the algorithms have been developed for the wild environment. However, those algorithms are built for specific defined conditions, adapt to limited variations, and can lead to low performance under some challenging wild environment conditions. In the future, researchers are working to develop new models to overcome the challenges of pose variation and occlusion and apply them practically in a complex real-world environment (Huang et al., 2019).

Recently, in computer vision, researchers focused on sex differences in facial expressions of emotion. Little research has been done on it, but it illustrates that females outperformed males in accuracy over males. In the coming research, researchers are focusing on intensity variations and sex differences in emotion recognition and sex differences will be considered while developing or proposing new models (Wingenbach, Ashwin & Brosnan, 2018; Cherbonnier & Michinov, 2021; Helgeson, 2020).

The security of life, assets, and data is the utmost concern. Face recognition biometrics and IoT technology-based security authentication have gained prominence. A face recognition-based door lock system has been developed. In the future, it can be extended to large buildings and organizations where more people are living. Furthermore, the Payroll system can also be integrated into the system for daily wage workers based on face recognition ID. For effectiveness and efficiency, the iris system can be integrated into the current system (Bagchi et al., 2022).

The smart city makes the traditional services for the inhabitants sustainable, efficient, and flexible through smart technology, smart healthcare, smart energy, smart transportation, and smart infrastructure. To make cities more responsive and efficient in the future, three emerging technologies, such as face recognition, the IoT, and cloud computing, can be appropriately integrated (Ibrahim Khalil & Kamel, 2024).

Intelligent face recognition devices are utilized in autonomous vehicles. A Face Recognition and Emotion Detection based on IoT (FRED-IoT) is proposed for tracking the driver’s face recognition and emotional state in autonomous vehicles. It can be more efficient if we collectively utilize visual and sensory knowledge for identification and managing multimodal production of knowledge in the future digital era (Chen, Feng & Zhang, 2022).

Recently, deep convolutional neural networks have achieved an accuracy of around 99% on the large dataset collected in the wild. Several studies demonstrated that the performance of deep learning models is recorded low in adverse situations, such as images with noise, blur, or illumination variations. Researchers have focused on developing deep learning models to be robust in adverse conditions (Wang & Deng, 2019; Grm et al., 2018; Pala & Erdem, 2019; Mohapatra et al., 2019).

The IoT technology greatly contributes to our daily lives in almost all sectors. Different IoT devices, such as cameras, are utilized in hospitals for surveillance scenarios. For the hospital’s security, face recognition is an important element to be used in patient traffic analysis, patient fraud detection, hospital security, emotions detection, and patient sentiment analysis. The utilized automatic intelligent face recognition systems work in a real environment, but the accuracy still needs to be improved in a controlled environment. Scientists are working on an intelligent face recognition system to enhance accuracy in real environments like smart healthcare (Masud et al., 2020).

Diagnosis of autism disease at the initial stages is very challenging as it is an abnormality in the brain. It can be diagnosed only through facial expression recognition because the facial patterns of autistic children are different from those of neurotypical children. Different face recognition-based systems have been developed based on six basic emotions. In the future, researchers will work to modify and enhance the existing systems for basic emotions and compound emotions to make them more effective and advanced (Talaat, 2023).

Integrating edge computing deep learning-based face recognition technologies can enhance data processing, instant decisions, and real-time processing with time delay. In facial expression recognition, edge computing technology is a “missing concept.” The researchers are focusing on integrating edge computing in complex emotion recognition systems. All devices are interconnected for information sharing, and real-time instant decisions are made on the edge with limited process power (Yang et al., 2021).

The 3D geometric details of the human face helped in 3D face recognition. It utilized a 3D sensor for the collection of data. The 3D facial model has improved facial expression recognition in the last ten years. Computer vision researchers have focused on resolving the issue of face recognition like makeup or beautification, pose, and sensitivity to light conditions (Adjabi et al., 2020).

In autonomous driving, the face recognition technology plays a vital role. In the future, the researchers have focused on making the system more intelligent by integrating an efficient recognition system. However, there are still numerous challenges at the infrastructure, architectural, algorithm, and system levels, which the researchers have focused on fixing (Gill et al., 2022).

Tremendous research works have been conducted on facial expressions of emotion to improve through the utilization of deep learning models. In the future, scientists are trying to develop an emotionally oriented deep-learning model by additionally integrating Internet-of-Things sensors to improve the recognition accuracy even for micro-expressions or compound emotions (Ko, 2018).

Huge research has been conducted on emotion recognition, but no standard data annotation rules exist. The commonly utilized annotation techniques are continuous and discrete, and there is no compatibility among datasets due to uniform standards for data annotation. For the description of different emotions, there is a need for different intensities. The currently available datasets are annotated with eight, six, or four emotions. The datasets are incompatible, and recognition models do not perform equally on these datasets. The researchers are trying to overcome the issues above by introducing a hybrid data annotation technique in which individual emotion is annotated according to its energy. While developing a hybrid data annotation technique, the researchers should care about selecting new data annotation standards because it may lead to imbalanced data. The biggest challenge in designing a model is generalizing it for new datasets or unseen data. It happened mostly due to no standard data-splitting techniques and limited sample data. Subject-independent and subject-dependent data splitting techniques have been used; the research shows that subject-independent techniques show better generalization. However, the subject-independent models are not more intelligent and cannot perform better in real-time and realistic applications. The researchers are trying to design subject-independent models that perform better in realistic, challenging environments (Ahmad & Khan, 2022).

The available existing datasets have limited samples or need to be more balanced. The deep learning models are data-hungry models that need huge datasets for better performance. Different augmentation techniques have been used to overcome overfitting issues. The datasets are required to capture the expressions from all possible angles for better realistic recognition, and the models should be efficient in real environments (Sajjad et al., 2023).

The education sector continuously evolves for an online connected environment by integrating the latest technologies. For future education models, diverse advanced technologies such as IoT, digital simulation tools, virtual reality, and artificial intelligence (AI) must collaborate. The researchers are working to make virtual or online learning management systems efficient by integrating the latest facial emotion recognition technology (Ahaidous, Tabaa & Hachimi, 2023).

### Definitions, trends and elements

### Definitions

**Emotion and basic emotion**: Emotion can be defined as the transient agitation produced due to joy, surprise, fear, and others (Suri & Gross, 2022). It can also be defined as an emotion, a complex physiological and psychological state produced in response to a stimulus, experience, or specific event (Cabanac, 2002). Descartes said that emotion is an important element used for communication and interaction among people and is a mediator between stimulus and response (Singh, 2012). The six basic emotion categories, such as surprise, sadness, happiness, fear, disgust, and anger, were first introduced by Ekman, Sorenson & Friesen (1969) and Ekman (1992).

**Compound emotion**: The compound emotion is the combination of two basic emotions simultaneously. For example, if a person is simultaneously happy and surprised, happy and surprised emotion features will appear on his face. There are 12 compound facial expressions of emotions, including disgustedly surprised, angrily disgusted, angrily surprised, fearfully disgusted, fearfully surprised, fearfully angry, sadly disgusted, sadly surprised, sadly angry, sadly fearful, happily disgusted, and happily surprised (Du, Tao & Martinez, 2014; Pendhari et al., 2022).

### Architectural elements of facial expression recognition system

Recognition systems, also known as pattern recognition systems, are designed to classify and identify features or patterns in data. These systems have been widely used in various fields, such as handwriting recognition, face recognition, speech recognition, and computer vision. The basic architectural elements of a face recognition system include face detection, preprocessing, feature extraction, and face recognition (Koutroumbas & Theodoridis, 2008; Rao & Reddy, 2011; Kasar, Bhattacharyya & Kim, 2016).

The face recognition process begins with the face detection step, where the input image is analyzed to determine the presence of face images. If face images are verified, the system proceeds to the preprocessing step, which involves the application of various techniques to remove unwanted effects such as shadowing, varying lighting, blur, and noise. Once the image has been cleaned and optimized, it moves on to the feature extraction phase.

During feature extraction, different algorithms are employed to extract important features from the image. This process involves noise cleaning, salience extraction, dimension reduction, and information packing. The facial patch is then transformed into a set of fiducial points along with their corresponding locations. These extracted features serve as the basis for the final step: face recognition.

In the face recognition step, the system performs automatic identification and verification based on the extracted features. Face recognition methods can be further classified into global feature-based and local feature-based approaches. Global feature-based methods consider the entire face as a single entity and extract features from the whole face, while local feature-based methods focus on specific facial regions and extract features from these localized areas.

**Figure 2:**
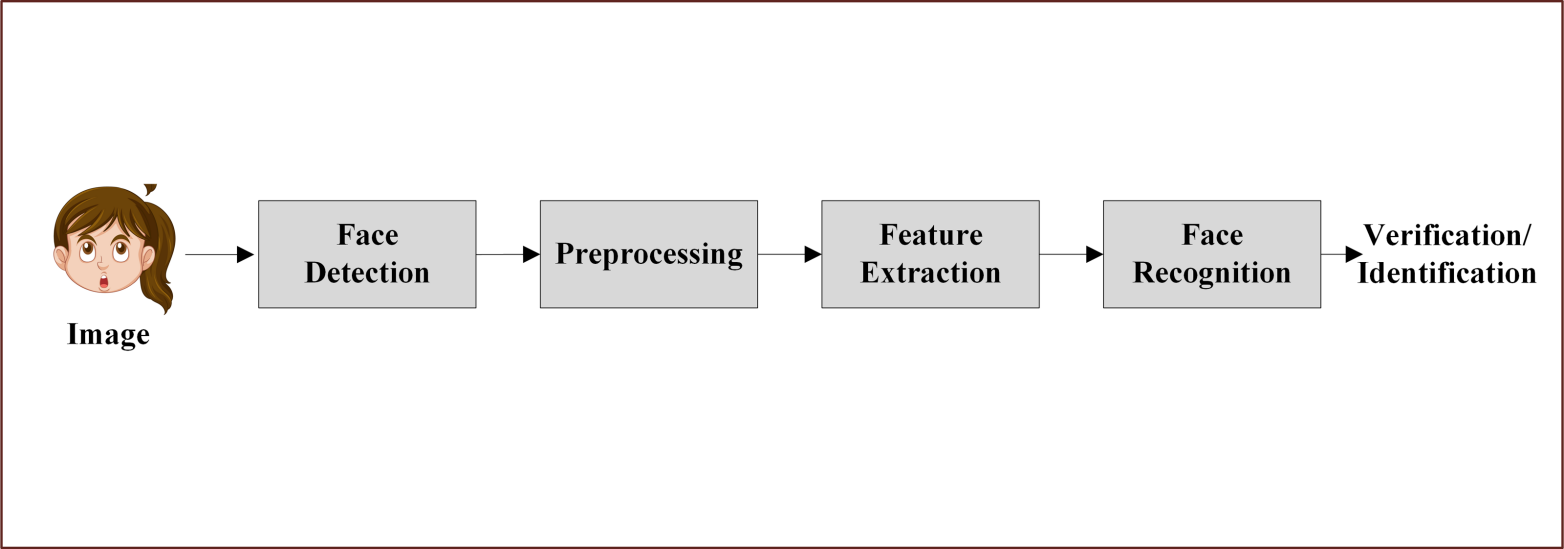
Architecture elements of FER system. Image source credit: Girl, Image by brgfx on Freepik (https://www.freepik.com/).

The architectural elements of a face recognition system work together to ensure accurate and efficient identification and verification of individuals. By following this structured approach, face recognition systems can be applied in a wide range of scenarios, such as security, surveillance, and access control. As research in this field continues to advance, we can expect to see further improvements in the accuracy, robustness, and versatility of face recognition systems (Rao & Reddy, 2011; Kasar, Bhattacharyya & Kim, 2016). Figure 2 shows the architectural elements of FER system.

### Trends

FER is a hot and trending topic in computer vision due to its tremendous applications in different fields. The applications of FER are not only limited to human–computer interaction (HCI) and computer vision, but it has diverse applications in different fields such as robotics and games, security, medicine, marketing, education, and many more. Intensity estimation and emotion recognition are the recent focus of the research (Ekundayo & Viriri, 2021). The popularity of different paradigms changes with time. We have searched Google search trends from 2004 till date for the Internet of Things, deep learning, facial expression, facial recognition systems, and machine learning from Google Trends. Recently, facial expression and facial recognition systems are gaining popularity (Google Trends, 2024).

### Applications

**Figure 3:**
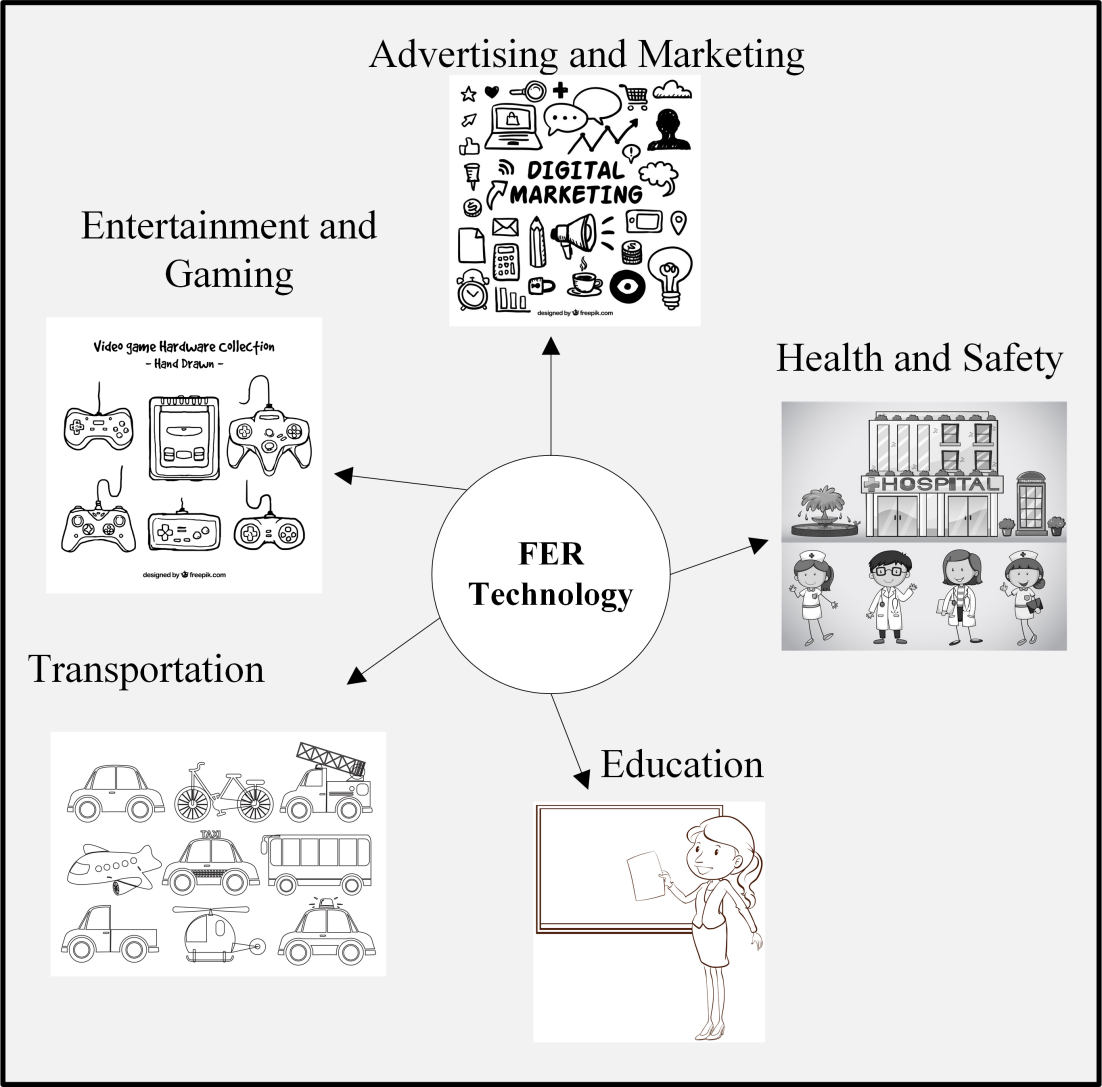
Applications of facial expression recognition. Image source credits: Doctors and nurses working at the hospital, Image by brgfx on Freepik; Digital marketing doodles, Image by Freepik; Collection of hand-drawn video game, Image by Freepik; Different kinds of transportations, Image by brgfx on Freepik; A plain sketch of a teacher, Image by brgfx on Freepik (https://www.freepik.com/).

Due to the rapid innovations and integration of facial expression recognition technologies, companies are rapidly growing worldwide. These companies have healthy competition due to the strong commercial potential of the applications of facial expression recognition technologies (Ali et al., 2021; Karnati et al., 2023). Figure 3 shows the different applications of facial expression recognition.

### Education

With the COVID-19 pandemic, traditional education has abruptly shifted towards online learning systems, presenting significant challenges. In face-to-face interactions, teachers can easily determine whether students are learning properly. The emerging facial expression recognition systems and diverse IoT devices, such as vision sensors and cameras, create an efficient student-teacher online interaction. Online learning is improved by integrating FER Enabled Education Aids IoT System, which can detect students’ emotional states and engagement levels, enabling personalized learning experiences (Geng, Meng & Dou, 2022). For efficient learning in distance learning and smart classrooms, a deep learning model is utilized based on facial expression recognition to detect the facial expressions of the students in real-time for student concentration analysis. This FER-based framework enabled teachers to create an efficient online learning environment (Hou et al., 2022; Wang et al., 2020).

### Advertising and marketing

Facial expression recognition systems have been used in marketing and customer experience. The FER system observes customers’ reactions to services, products, and advertisements, which enables the companies to build the best marketing strategies based on customer feedback (Van Thanh, 2023). Facial expression recognition architectures are used in diverse emotions recognition which the market researchers used for customer feedback analysis and sentiment analysis for planing future marketing and business strategies of companies (Srivastava & Bag, 2023; Van Thanh, 2023).

### Entertainment and gaming

Facial expression recognition technology can also be utilized in games and the entertainment sector. It is embedded in virtual reality and augmented reality applications-based games for an extensive and realistic experience. It can be utilized in social media platforms for relevant content recommendations based on the collected information (Ansari et al., 2022). FER in virtual reality (VR) revolutionized the entertainment sector. In VR games, a dynamic world is created to respond according to the player’s movements and facial features. The FER revolutionized the entertainment industry by delivering engaging and unique experiences. FER technology is also used in getting audience feedback from facial expressions (Zhang & Fort, 2023).

### Health

Facial expression recognition algorithms can identify a wide range of emotions, such as surprise, anger, sadness, and happiness. Mental health professionals can utilize these algorithms to monitor patients’ emotional well-being. Experts in mental health monitoring, customer feedback analysis, and market research can utilize it (Srivastava & Bag, 2023). The FER system is employed in patient health monitoring. It detects the patient’s pain or abnormal condition based on facial features and informs professionals about on-time treatment and health monitoring (Van Thanh, 2023).

### Transportation

FER is one of the most powerful signals of human beings for conveying emotional state. For real-time monitoring of the driver, FER is integrated for detecting drowsiness, fatigue, distraction, or other unfavourable emotional state which affect driving performance. It significantly decreased accidents as it timely alerted drivers (Xiao et al., 2022). A facial expression recognition-based system is developed called Vibraimage. It detects micro-facial expressions of a passenger in an airport station to declare a passenger suspect or non-suspect. If a person due to some accidental facial expressions is declared as suspected by the system then he will be passed from screening. It significantly improved transportation security (Wright, 2023).

### Facial expression recognition models/ frameworks

For the last three decades, facial expression recognition has been one of the prominent research areas in image processing, artificial intelligence, and machine learning communities. Diverse recognition frameworks and models are proposed for the recognition and classification of facial expression recognition. Classical approaches were the initial models introduced around the 1950s. The 2D features were extracted from photographs, and These methods worked on geometrical relationships among facial points. The methods faced many challenges but they provide a basis for advanced face recognition technologies research. Another face recognition system was developed by Kanade (1973). It utilized traditional image processing procedures to extract a vector of 16 facial parameters (Kanade, 1973; Roberts, 1963; Zhao et al., 2003).

The holistic methods are also called global features-based methods. It performs recognition based on global characteristics. It focuses on the complete face for recognition and not on individual components of the face, such as the nose, eyes, and mouth. The global features extraction was first utilized by Sirovich & Kirby (1987) in principal component analysis (PCA). It was used in independent component analysis (ICA), subspace linear discriminant analysis (SLDA), and linear discriminant analysis (LDA). Later on, another method called support vector machine (SVM) was introduced to recognize emotions (Bartlett, Movellan & Sejnowski, 2002; Turk & Pentland, 1991a; Turk & Pentland, 1991b; Etemad & Chellappa, 1997; Zhao & Chellappa, 1999; Guo, Li & Chan, 2000).

The hybrid methods are the concatenation of holistic and local methods simultaneously. It is a common trend in research work and utilizes the strengths of the combined methods for improved performance. Tolba (2000) proposed a method that was the hybrid of learning vector quantization (LVQ) and radial basis function (RBF). Lu, Wang & Jain (2003) extended it by the combination of LVQ, RBF, and local binary patterns (LBP). Lavanya & Hannah Inbarani (2018) proposed a method combining tolerance rough similarity (TRS) and PCA. TRS is utilized for extracting similarity index, and PCA for extracting feature matrix (Gutta & Wechsler, 1997; Lu, Wang & Jain, 2003; Tolba, 2000; Hashemi & Gharahbagh, 2015; Hu et al., 2017).

With the advances in computer vision, different new models have been developed in the modern world of emotion recognition. The modern approaches are categorized into fuzzy logic-based methods, dictionary learning-based methods, and deep learning-based methods. Machine learning methods such as k-nearest neighbors (kNN), artificial neural networks (ANNs) including multi-layer feedforward networks, logistic regression, decision trees, and SVMs are used for classification after the feature extraction phase. These methods have achieved moderate success in their use. Many studies have utilized this classification pipeline of hand-crafted features and popular classifiers to perform classification (Rincy & Gupta, 2020; Barata et al., 2014; Blum et al., 2004; Rastgoo et al., 2015).

Transfer learning (TL) is regarded as a machine learning technique where information obtained from one task is applied to a specific task to enhance learning. TL performs efficiently in distinct feature areas or information patterns when huge data samples are available for training. Inferential TL, unsupervised TL, and supervised TL are three major categories for TL with different subsections. TL techniques using a pre-trained model and convolutional network are the most common strategies in object detection and classification. A pre-trained model is developed using huge benchmark datasets such as ImageNet, comprising enhanced feature attributes at all levels of abstraction. Simply by incorporating new convolutional or dense layers in a pre-trained model as a feature extractor and then fine-tuning the new model *via* continuous backpropagation, these feature representations may be partially or wholly reused in subsequent tasks. Numerous applications have demonstrated that this method may be used to create cutting-edge object detectors (Yu, Xiu & Li, 2022; Ribani & Marengoni, 2019; Ebrahim, Al-Ayyoub & Alsmirat, 2019).

Machine learning is a field that is raised out of artificial intelligence (AI). It involves using statistical models to automatically learn patterns from data without defining the patterns explicitly. Machine learning is defined as “the automated detection of meaningful patterns in data”, meaning programs learn to perform simple and complex tasks after gaining more experience from past information. There are a lot of practical applications of machine learning ranging from computer vision applications (such as face detection, image retrieval, *etc*.), speech processing (such as speech synthesis, language modeling, *etc*.), natural language processing (NLP) (such as context-free parsing, named-entity recognition, *etc*.) and other problems including fraud detection, *etc* (Shalev-Shwartz & Ben-David, 2014; Murdoch et al., 2019).

Another renowned neural network is the RNN, which uses sequential or time-series data and feeds the results of the prior stage as input to the current stage. Recurrent networks, such as feedforward and CNN, learn from training input, but they stand out due to their “memory”, which enables them to influence current input and output by drawing on data from earlier inputs. The output of an RNN depends on previous components in the sequence, in contrast to a typical DNN, which presumes that inputs and outputs are independent of one another. This feature is essential to a variety of applications since the embedded structure in the data sequence provides valuable information (Dupond, 2019).

Deep learning is another field of machine learning in artificial intelligence concerned with algorithms capable of learning from data. The term deep learning simply means training neural networks or deep neural networks to learn a general representation of data. Furthermore, deep learning aims to estimate a function, f ∗. For example, all a deep learning classifier y = f(x) does is to map an input x to it corresponding category y .Given a deep learning network, there is a defined mapping y = f(x; *θ*) and appropriate value of parameters that needs to be learned in order to get the best estimation function. In, their deep learning model outperforms human performance, which shows the promising future of deep earning algorithms for computer vision tasks. Deep learning usually consists of several hidden layers, while shallow neural networks are made up of fewer hidden layers. There have been many variant forms of deep learning architectures used to solve a lot of diverse problems in fields such as computer vision, natural language processing and speech recognition. Some of these architectures are deep CNN, RNN and deep belief networks. Recently, with the recent availability of large amount of data and computational power, deep learning gained more attention (He et al., 2016; Szegedy et al., 2015).

### Facial expression recognition, Internet of Things, and cloud computing

In recent years, the IoT technology has been integrated into various application domains, including transportation, video surveillance, healthcare, education, and many more, to enable object/device connectivity and data sharing. A smart city comprises different components such as cloud servers, wireless networks, smart healthcare, smart shopping, smart traffic systems, and smart homes. Smart devices and IoTs capture different signals, including facial expressions, and send them to the cloud server for processing and decision-making. The decisions are then forwarded to the stakeholders for necessary actions (Masud et al., 2020; Yassine et al., 2019).

Cloud computing (CC) is an emerging technology with ubiquitous characteristics such as seamless accessibility, high scalability, and online storage, ultimately reducing workforce and capital costs. It has attracted organizations to run their financial activities and businesses over the cloud. However, despite its strengths, cloud computing has some drawbacks in terms of security concerns (Tahirkheli et al., 2021). Facial expression recognition technology, when combined with the IoT and cloud computing, has been integrated into autonomous driving systems for monitoring drivers’ emotional states. Intelligent and automated facial expression recognition devices are more efficient in detecting the emotional states of the driver, enabling real-time safety measures and personalized driving experiences (Chen et al., 2021).

Integrating edge computing into IoT devices can make facial expression recognition algorithms more convenient and faster, improving sustainability and decreasing costs. The development of action unit (AU) based expression recognition algorithms for edge devices has enhanced processing time by eliminating the intermediate transfer process (Yang et al., 2021).

Integrating physical equipment into facial expression recognition systems can improve efficiency, productivity, and services. For example, numerous IoT-enabled vehicle recognition systems have been developed in which UHF-RFID windshield tags are integrated for efficient recognition (Mufti et al., 2023). These systems can be extended to incorporate facial expression recognition, enabling emotion-aware intelligent transportation systems.-wildlife conflict (HWC) is one of the critical global conflicts. A deep convolutional neural network (DCNN) based recognition system has been developed, which captures images of animals, saves them in .jpg format, and sends the images to the base station *via* a radio frequency (RF) network. It classifies and extracts features from the images, and whenever an animal is identified, it sends a message alert. In such systems, IoTs play a significant role and can be implemented by forest officials for the safety of people and wildlife (Surya & Selvaperumal, 2022). Similar systems can be adapted for facial expression recognition to detect and respond to human emotions in wildlife conservation efforts.

With the COVID-19 pandemic, traditional education has abruptly shifted towards online learning systems, presenting significant challenges. In face-to-face interactions, teachers can easily determine whether students are learning properly. The emerging facial expression recognition systems and diverse IoT devices, such as vision sensors and cameras, create an efficient student-teacher online interaction. Online learning can be improved by applying FER Enabled Education Aids IoT System, which can detect students’ emotional states and engagement levels, enabling personalized learning experiences (Geng, Meng & Dou, 2022).

**Figure 4:**
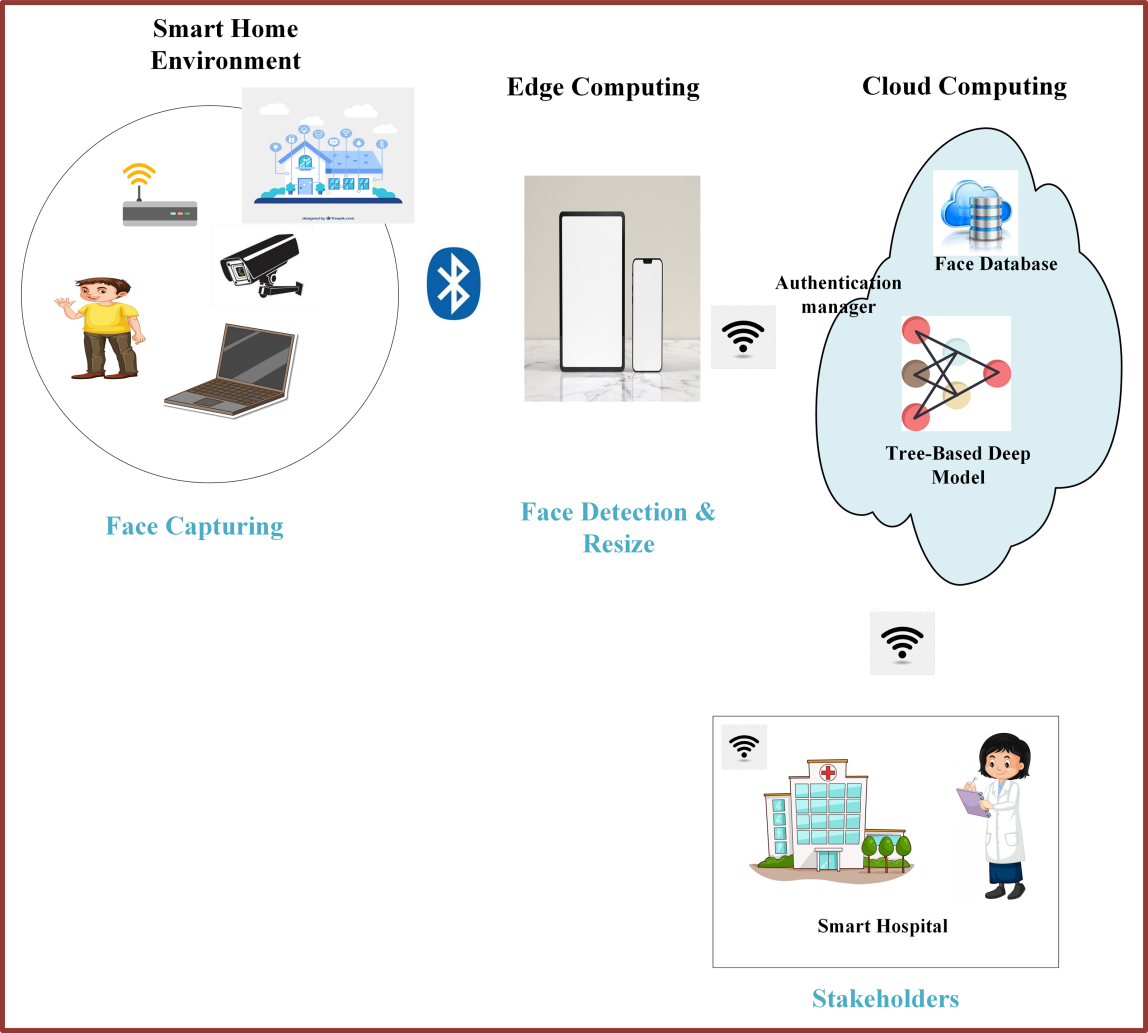
Smart city framework. Image source credits: Smart home, Image by Freepik; Boy in yellow shirt, Image by brgfx on Freepik (https://www.freepik.com/). CCTV, https://www.rawpixel.com/image/6755502/png-sticker-public-domain, CC0; Bluetooth, Hospital, https://clipart-library.com/clipart/36700.htm, Non commercial use only; Devices on marble table, Image by Freepik; A sticker template with a laptop isolated, Image by brgfx on Freepik; Wifi Icon Vector, Image by iyikon on vecteezy.com; Wifi icon, Image by seetwo on vecteezy.com; Cloud computing concept, Image by macrovector on Freepik; Learning Deep Algorithm Data, Image by flatart on vecteezy.com; Girl in science gown writing note on white, Image by brgfx on Freepik.

The advent of facial expression recognition technology and the IoT technology is making human life smarter and more efficient. For example, the door of an apartment can recognize the owner’s facial expressions and open automatically through smartphone activation, enhancing security and convenience. Additionally, this technology can help in identifying lost people and criminals by analyzing their facial expressions (Medapati, Murthy & Sridhar, 2019). The smart city is illustrated in Fig. 4.

### Open challenges

With the tremendous contributions of face recognition, there are still challenges faced by real-world face recognition-based systems that need to be addressed. Most challenges are faced in an uncontrolled environment, where subjects are dynamic, and variations are difficult to catch in machine perspectives due to certain causes. Different challenges to face recognition have been discussed below in detail.

### Lab-made compound facial expressions

In some situations, Ekman’s basic emotions cannot represent human emotions. For example, at a surprise birthday party, the individual simultaneously feels a mixture of happy and surprised emotions. Du & Martinez (2015) proposes new compound emotions classes, combining basic emotions such as angrily and happily surprised. Du, Tao & Martinez (2014) images of different compound emotions were collected by recruiting diverse volunteers, and the images were taken by training the volunteers about the specific scenario in a controlled manner to act accordingly for each compound emotion. This approach is called ‘lab-made facial expressions.’ The images collected are captured based on the individual capability and simulated according to the specific direction. It is a challenge as such expressions are rare in real physical environments. The current compound facial expression image datasets may not express internal human emotion, so the compound emotions images can be collected from a real environment and should represent an individual’s real compound emotions and feelings (Du & Martinez, 2015; Du, Tao & Martinez, 2014; Win, Siritanawan & Kotani, 2023; Parent, 2005).

### Pose variation

The pose variation means the rotation of the subject image in diverse 3D or 2D perspectives. It is challenging when a suspect, terrorist, or thief is in the airport or public crowd. Identifying suspects in public crowds with different poses is difficult, especially for law enforcement agencies. In a stored passport database, a single photo cannot correctly identify a suspect in a crowd with different poses. So, pose variation issues need to be addressed by researchers in the future (Zhang, Zhang & Huang, 2013).

### Illumination variation

Illumination variation is another serious challenge for face recognition systems in real-world face recognition-based systems. The illumination and environmental conditions are not under the control of machine intelligence. Tradition machines and deep learning models cannot address the illumination variation (Zhao & Chellappa, 1999).

### Occlusion

Occlusion is a crucial challenge for computer vision scientists in real-world face recognition systems. In occlusion, the important features of the face are hidden due to the presence of other objects, such as a mask, scarf, hand, sunglasses, helmet, or hat. Tremendous research has been done on it, but it can still be enhanced (Gautam & Mukhopadhyay, 2019; Guo et al., 2017; Shahid, 2023).

### Aging

Aging is the most challenging for face recognition systems as, with age, the features of the face are changing continuously, and it is difficult for intelligent machines to recognize faces with varying aging effects. It is a harder challenge than other face recognition challenges, and few efforts are available in the literature to address the aging issue of face recognition systems (Fu, Guo & Huang, 2010).

### Lack of compound facial expression publicly available datasets

There are different challenges and limitations concerning recognizing compound expressions of emotion. The available databases for recognizing compound expressions of emotion could be more extensive. Furthermore, the publicly available datasets contain a very small number of categories, 23 (EmotionNet) and 22 (CFEE), which cover a small portion of conceivable compound emotions. Furthermore, it requires a large amount of training data, especially in quality and quantity training deep neural networks. Currently, the datasets utilized are of a limited number of categories and unbalanced data distributions (Benitez-Quiroz, Srinivasan & Martinez, 2016; Benitez-Quiroz et al., 2017; Li & Li, 2020).

### Research methodologies issues

The running techniques concerning complex emotion profiling have limitations concerning detection and need a large dataset for the emotion profiling model. Other findings show that compound emotions have yet to be analyzed deeply. Applying deep learning methods requires a large and balanced training dataset for optimal performance. For the recognition of compound expressions of emotion, diverse traditional machine learning approaches are utilized, like SVM and kNN, others, but the utilized traditional methods have different limitations; for the manual tuning of parameters, there is need of more labor work. Another area for improvement is the incapability of detecting images, which is performed spontaneously and uncontrolled. It is argued that most of the recognition of compound expressions of emotion has been done through databases and is based on algorithms and unbalanced classifications, which ultimately lead to a lack of precision. Compound emotion labeling is performed automatically through machine learning-based algorithms, which leads to inaccuracies. The main cons of using the kNN algorithm is the utilization of whole features equally in the field of computing for similarities, which ultimately leads to classification errors (Jarraya, Masmoudi & Hammami, 2020; Thuseethan, Rajasegarar & Yearwood, 2020; Guo et al., 2018).

### Recognition of limited compound emotions

The compound expressions of emotions are not explored due to their complexities. Compound emotions are mainly created by combining basic emotions. In some methods and databases, it can only recognize a limited number of compound expressions of emotion. Currently, the datasets utilized are of a limited number of categories and unbalanced data distributions (Liliana, Basaruddin & Widyanto, 2017; Liliana, Basaruddin & Oriza, 2018).

### Computational time issue

The recognition of compound facial expressions of emotion is a critical job in large databases. For the recognition of specific image in large databases, diverse algorithms have been implemented. In addition, it takes more computational time and space for dealing with large databases (Taskiran, Kahraman & Erdem, 2020).

### FER in video surveillance

FER-based recognition systems are becoming common and have varied applications, especially video surveillance. In this setting, the performance of FER systems is mostly reliant on image acquisition conditions, mainly when the posture changes, and because the acquisition techniques may include artifacts. We mainly discuss camera focus problems that can lead to image blurring, low-resolution, or compression-related errors and block effects. In this case, the challenge of FER systems is to distinguish individuals from photographs captured employing video surveillance cameras, presenting blurred, low-resolution, block artifacts or faces with variable poses. This challenge remains an unsolved problem and requires further research (Zhang, An & Zhang, 2018).

### Facial expression recognition and smartphones

Adopting FER on mobile devices offers many advantages. In addition to the employment facility, the users do not have to remember the PIN or password; it can be conveniently implemented on tablets and smartphones because only the frontal camera is required. FER systems have been used in recent years to secure devices and control access to many different services through smartphones, such as purchases or online payments on the store. While adopting FER systems on smartphones provides many advantages, many challenges must be addressed. The user’s facial image should be captured in a comfortable or constrained environment. Many factors, such as pose and ambient lighting due to various ways of interacting with mobile technology and imaging distance, can restrict facial image quality (Adjabi et al., 2020).

### Cross-cultural variability

Emotional expressions vary considerably from culture to culture, region, and language. It is very challenging for researchers to develop robust models to overcome the cross-cultural variability in expressions of emotion. New robust models need to be developed to integrate and consider cross-cultural variability. Furthermore, it is more challenging in the case of compound facial expressions of emotions (Vashishth et al., 2023).

### Multimodal challenges

Multimodal machine learning-based models are also utilized for emotion recognition, integrating diverse modalities such as facial expressions, speech, text, and others. Due to its complexity due to different modalities, It is challenging for the model to extract relevant important features from each modality appropriately (Liang & Morency, 2023).

### Low resolution

Research shows that facial recognition models have achieved an accuracy of 99.63% on high-resolution quality images, but low-resolution image datasets for automatic facial expression recognition are a challenge. Despite remarkable research on developing diverse models for high-resolution images, little attention is given to low-resolution images. The low-resolution issue mostly occurred to models in real-life environments, as the image captured in a real environment will have the issue of low resolution, illumination, and pose variation (Lo et al., 2023; Cheng, Zhu & Gong, 2019; Massoli, Amato & Falchi, 2020).

### Ethnic differences

In the 21st century, numerous technologies have been utilized to deal with emotion recognition with key breakthroughs. The facial expressions of emotion recognition systems face tremendous issues, but ethnic differences are one of the critical hurdles. Charles Darwin also mentioned that emotional expressions are invariant and can be changed from one ethnic group to another for the same facial expression of emotion. It is a challenge for diverse experimental, methodological, and theoretical grounds (Ahmad et al., 2022; Costantini et al., 2022).

### Future directions

With the innovations in computer vision, numerous advanced deep learning models have been developed to recognize facial expressions of emotions. However, researchers face diverse challenges concerning emotion recognition. While computer vision scientists have overcome various challenges, new challenges continuously arise with new environmental situations and the installation of recognition systems in complex environments. In this section, we discuss different challenges that need to be addressed in future research and explore their implications for the field.

### New models for compound emotions recognition

Compound emotions, also called mixed emotions, blend emotions with complex features compared to basic emotions. Accurately recognizing each class of compound emotions is challenging for existing deep learning models due to the complexity in feature extraction and the difficulty in differentiating between complementary and dominating emotions. Future research should focus on developing novel dedicated models specifically for compound facial expressions of emotions. Collaboration among computer scientists, psychologists, neuroscientists, linguists, and social scientists can lead to more comprehensive and accurate emotion models. Addressing this challenge will significantly advance the field of emotion recognition, enabling more nuanced and context-aware applications in various domains, such as healthcare, education, and human–computer interaction (Wagner, 2019; Zhang et al., 2020).

### New cross cultural adoptive models

Facial expressions vary across genders, cultures, and ages. An ideal database and cross-culture adaptive model is necessary to account for these differences. Future research should focus on developing a new cross-culture-adaptive model that efficiently recognizes basic and compound emotions while considering cross-cultural differences. This will require the creation of datasets that include all basic and compound facial expressions of emotions with appropriate attribute labels, ethnicity, gender, and age. Addressing this challenge will enable the development of more inclusive and equitable emotion recognition systems, with applications in fields such as marketing, advertising, and social robotics (Jack et al., 2012).

### Multi-pose facial recognition model

Pose variation is a significant challenge for face recognition systems, particularly in crowded environments where individuals continuously change poses. Future research should focus on developing novel models that can effectively handle pose variations. Potential solutions include the use of 3D face modeling, pose-invariant feature extraction, and multi-view face recognition techniques. Overcoming this challenge will significantly improve the performance and reliability of face recognition systems in real-world scenarios, such as surveillance, security, and law enforcement (Ding & Tao, 2016).

### Enhancing dataset for compound emotion recognition

To advance research on cross-cultural, cross-gender, and cross-age range facial expression recognition, there is a need for enhanced datasets that include high-resolution images, more compound emotion categories, and multi-pose and occlusion annotations. Creating such datasets through collaboration among multi-field experts, including computer scientists, psychologists, neuroscientists, linguists, and social scientists, can lead to more comprehensive and accurate datasets that are universally acceptable. Addressing this challenge will provide a solid foundation for the development of more robust and generalizable emotion recognition models (Karnati et al., 2023).

### Efficient novel models for low and high resolution images

Most deep learning models perform efficiently on high-resolution images but struggle with low-resolution images, which are common in real-time recognition systems. Future research should focus on developing new models that can efficiently handle both low and high-resolution images, making them more adaptable and efficient for real-time recognition-based systems. Potential solutions include the use of super-resolution techniques, multi-scale feature extraction, and attention mechanisms. Overcoming this challenge will enable the deployment of emotion recognition systems in a wider range of applications and environments (Lo et al., 2023).

### Novel models based on merging human brain emotion perception

Integrating advanced imaging techniques, such as functional Magnetic Resonance Imaging (fMRI), into emotion recognition models can provide insights into real-time brain changes during emotion perception. By linking brain neuronal activity to emotion recognition, future models can achieve better performance and incorporate additional contextual behavioral cues. This interdisciplinary approach, combining neuroscience and computer vision, has the potential to revolutionize emotion recognition and deepen our understanding of human emotions. Addressing this challenge will require close collaboration between researchers in both fields and the development of novel algorithms that can effectively integrate fMRI data into emotion recognition models (Tipirneni & Leal, 2022).

### Advance context-based models

While significant research has been conducted on facial expression recognition, current models often struggle to accurately trace the real feelings of users in context. Future research should focus on developing emotion recognition models that can recognize an individual’s real feelings in relation to the context. This will require the development of advanced artificial intelligence-based systems that can integrate multiple modalities and optimize architectures for real-time systems. Addressing this challenge will enable the creation of more empathetic and context-aware emotion recognition systems, with applications in fields such as mental health, customer service, and social robotics (Cîrneanu, Popescu & Iordache, 2023).

### Gait based intelligent classification model

Gait-based emotion recognition, which interprets and analyzes emotions based on walking patterns, is an emerging area of research. Deep learning-based models have yet to be extensively utilized in this domain, presenting an opportunity for future research. The fusion of different deep neural networks for gait-based emotion recognition could lead to significant breakthroughs. Addressing this challenge will expand the scope of emotion recognition beyond facial expressions, enabling the development of more comprehensive and multi-modal emotion recognition systems (Xu et al., 2022).

### Suppression of noise labels

Databases of wild conditions often contain noisy and subjectively labeled data due to occlusion and illumination issues, leading to lower performance of facial expression recognition models. Future research should focus on developing techniques for noisy label suppression to overcome this challenge (Shao & Luo, 2021). Potential solutions include the use of unsupervised or semi-supervised learning, data augmentation, and robust loss functions. Addressing this challenge will improve the reliability and generalizability of emotion recognition models in real-world scenarios.

## Conclusions

Significant research has been conducted on FER based systems. It is utilized in diverse fields, and computer vision scientists constantly encounter new advancements and challenges. In this article, we have discussed the basics of FER-based systems, architectural elements, FER applications, FER-based global leading companies, Interconnection between FER, IoT, and cloud computing, Open challenges to facial expression recognition technologies, and future directions. Overcoming the identified challenges and future directions in this research through researchers in the future will bring evolution in the field of computer vision.

## Additional Information and Declarations

### Data Availability

The following information was supplied regarding data availability: This is a literature review.
